# Three‐dimensional bone morphology is a risk factor for medial postmeniscectomy syndrome: A retrospective cohort study

**DOI:** 10.1002/jeo2.12090

**Published:** 2024-07-21

**Authors:** Jonas Grammens, Annemieke Van Haver, Femke Danckaers, Kristien Vuylsteke, Jan Sijbers, Lotem Mahluf, Peter Angele, Elizaveta Kon, Peter Verdonk, MEFISTO WP1 Group

**Affiliations:** ^1^ Antwerp Surgical Training, Anatomy and Research Centre University of Antwerp Wilrijk Belgium; ^2^ imec‐VisionLab, Department of Physics University of Antwerp Wilrijk Belgium; ^3^ More Institute Deurne Belgium; ^4^ Active Implants BV Haarlem Netherlands; ^5^ Clinic for Trauma and Reconstructive Surgery University Hospital Regensburg Regensburg Germany; ^6^ Sportopaedicum Regensburg Regensburg Germany; ^7^ Humanitas Clinical and Research Center ‐ IRCCS Rozzano Milan Italy; ^8^ Department of Biomedical Sciences Humanitas University Pieve Emanuele Milan Italy; ^9^ Department of Orthopaedics University Hospitals Antwerp Edegem Belgium; ^10^ OrthoCA Antwerp Belgium

**Keywords:** 3D, knee, meniscectomy, morphology, predictive model

## Abstract

**Purpose:**

The study aims to identify differences in tibiofemoral joint morphology between responders (R group, no pain) to arthroscopic partial medial meniscectomy (APMM) versus medial postmeniscectomy syndrome patients (MPMS group, recurrent pain at 2 years postmeniscectomy) in a clinically neutrally aligned patient population. The second aim was to build a morphology‐based predictive algorithm for response to treatment (RTT) in APMM.

**Methods:**

Two patient groups were identified from a large multicentre database of meniscectomy patients at 2 years of follow‐up: the R group included 120 patients with a KOOS pain score > 75, and the MPMS group included 120 patients with a KOOS pain score ≤ 75. Statistical shape models (SSMs) of distal femur, proximal tibia and tibiofemoral joint were used to compare knee morphology. Finally, a predictive model was developed to predict RTT, with the SSM‐derived morphologic variables as predictors.

**Results:**

No differences were found between the R and MPMS groups for patient age, sex, height, weight or cartilage status. Knees in the MPMS group were significantly smaller, had a wider femoral notch and a smaller medial femoral condyle. A morphology‐based predictive model was able to predict MPMS at 2 years follow‐up with a sensitivity of 74.9% (95% confidence interval [CI]: 74.4%–75.4%) and a specificity of 81.0% (95% CI: 80.6%–81.5%).

**Conclusion:**

A smaller tibiofemoral joint, a wider intercondylar notch and smaller medial femoral condyle were observed shape variations related to medial postmeniscectomy syndrome. These promising results are a first step towards a knee morphology‐based clinical decision support tool for meniscus treatment.

**Study Design:**

Case–control study.

**Level of Evidence:**

Level IIIb.

**Abbreviations**
3D: three‐dimensional
AP: anteroposterior
APMM: arthroscopic partial medial meniscectomy
AUC: area under the curve
BMI: body mass index
CI: confidence interval
IKDC: International Knee Documentation Committee
KOOS: Knee injury and Osteoarthritis Outcome Score
ML: mediolateral
MPMS: medial postmeniscectomy syndrome
MRI: magnetic resonance imaging
n.s.: nonsignificant
OA: osteoarthritis
PACS: picture archiving and communication system
PC: principal component
PCA: principal component analysis
PD: proton density
PROM: patient‐reported outcome measure
RMSE: root mean square error
ROC: receiver operating characteristic
RTT: response to treatment
R group: responder group
SD: standard deviation
SSM: statistical shape model

## INTRODUCTION

Among possible surgical treatment options for medial meniscal tears, arthroscopic partial medial meniscectomy (APMM) is one of the most regularly performed knee surgery worldwide [7, 18, 24, 25, 46]. Recent insights have resulted in an evolution of its indications [38], and there is a paradigm shift towards preserving the meniscus to the greatest extent possible [1].

Current guidelines [6, 26] do not recommend APMM as a first‐line treatment but prefer meniscal repair or conservative treatment instead. It might, however, serve as an alternative when the latter two treatment options are not applicable (complex tears, tears with high degree of degeneration, flap tears or nonreducible bucket handle tears) or when response to meniscal repair or conservative treatment has been unsatisfactory.

A significant subset (6%–25%) of partial meniscectomy patients experience persistent or recurrent pain within 1–2 years after APMM [13, 49], also known as the postmeniscectomy syndrome. These patients typically suffer from dull and nagging pain in the operated knee compartment, often accompanied by transient joint effusions [37].

Several studies have established common risk factors for inferior APMM outcome [42], including age, obesity, cartilage status, coronal malalignment [48] and the extent of the meniscectomy [14]. These risk factors are all related to a mismatch between the applied load (obesity, activity level, coronal malalignment) and reduced resilience to resist and endure that load (meniscus dysfunction or cartilage loss).

An often overlooked but potentially significant risk factor for medial postmeniscectomy syndrome (MPMS) is three‐dimensional (3D) knee morphology. Previous publications on medial knee compartment morphology indicated a potential link between a small medial femoral condyle and early degeneration of the medial meniscus [17, 44]. The relationship between specific bony knee morphology variations and certain pathologies such as cruciate ligament lesions [15] and patellar instability [20] has already been demonstrated. Although bony morphology is nonmodifiable, it is of great importance to detect and acknowledge this factor, as it may have an influence on surgery outcomes [36].

A commonly used method to analyse bone morphology is measuring several anatomical landmark‐based distances and angles, either on the 3D bone models or directly on the medical images. While very straightforward and easy to visualise, this approach suffers from some limitations. First, it only captures information at discrete, predefined anatomical landmarks, thereby neglecting the complexity of shape variation patterns over the entire bone surface. Second, the choice of morphological parameters or measurements is subjective and might be prone to a selection bias, potentially overlooking essential morphological features.

Statistical shape modelling offers a powerful alternative that overcomes these limitations. By analysing large data sets of 3D bone models (e.g., from magnetic resonance imaging [MRI] scans), statistical shape modelling involves the creation of a smart shape atlas that captures the average bone shape and its main modes of shape variation in a data‐driven way. It eliminates the need for subjectively chosen landmarks and instead analyses the entire articular bone surface at once. As a result, it will lead to a deeper understanding of the complex interplay between distinct morphological features in the context of APMM outcome.

Statistical shape modelling has been around for several years [11] but only recently found its way to the field of orthopaedics [10, 19, 29, 32]. In the present study, it allows to quantitatively compare the femoral and tibial bone shapes between two groups of meniscectomy patients and to extract potential morphological predictors for clinical response to APMM.

The purpose of this study was to investigate if adult APMM patients who develop pain symptoms (MPMS) demonstrate different bony knee morphology, compared to APMM patients who do not develop pain symptoms, within a follow‐up of 2 years after APMM. Based on our previous findings [17, 44], related to a small medial femoral condyle morphotype as a risk factor in the multifactorial process of medial compartment knee pain, we hypothesise the MPMS knees to have a smaller medial femoral condyle. Finally, this study aimed to evaluate a predictive model for APMM outcome with bony morphology as predictor variables.

## MATERIALS AND METHODS

This study is a multicentre, retrospective case–control study. Three high‐volume orthopaedic centres specialised in knee pathology from Antwerp, Milan and Regensburg participated in this study. Institutional Review Board approval was obtained from all local ethical committees (Comité voor medische ethiek AZ Monica and UZA: study B300201941743, Ethikkommission an der Universität Regensburg: reference 19‐1621‐101, IRCCS Instituto Clinico Humanitas: study authorisation n. 2515), and informed consent was obtained from all patients prior to their inclusion.

### Patient selection and study design

Patients were eligible for the study if they were between 18 and 70 years old and had a primary medial meniscus lesion, for which meniscal repair or conservative treatment was not applicable, and, hence an APMM was indicated and performed by an expert surgeon (*N* = 844). Predefined exclusion criteria were unavailable preoperative MRI, inability to communicate, an unstable knee (International Knee Documentation Committee [IKDC] grade C or D), patellar instability or trochlear dysplasia, limited knee range of motion (IKDC grade C or D), cartilage lesions (grade IV and larger than 2 cm, nonfocal), coronal malalignment (as judged clinically), concomitant discoid meniscus, morbid obesity (body mass index [BMI] > 35), a history of meniscus repair or major lower limb surgery prior to the meniscectomy, septic or rheumatoid arthritis, neurological disorders, posterior cruciate ligament repair or reconstruction, insufficiency fractures or avascular necrosis, plica syndrome or less than 2 mm intact medial meniscal rim left intraoperatively. Finally, 443 patients were eligible after screening of their hospital records, of which 42 had an MRI of insufficient quality (movement artefacts or slice thickness > 4 mm) and 161 did not consent study participation or did not complete the Knee injury and Osteoarthritis Outcome Score (KOOS) questionnaire.

As pain is the primary symptom for diagnosis of the postmeniscectomy syndrome, the KOOS pain subscore [40, 41] was used to split the APMM patients into two groups: a first group who showed a good clinical response to APMM (further referred to as R group) and a second group who developed MPMS (further referred to as MPMS group). A power analysis defined the required sample size as 120 patients per group. The KOOS pain score threshold for stratification of the subgroups was set at 75, based on the patient acceptable symptom state, as calculated by Agarwalla et al. [2]. The R group included 120 patients with a KOOS pain score > 75 and the MPMS group included 120 patients with a failed clinical outcome, defined as KOOS pain score ≤ 75. The predefined total study sample size (*N* = 240) was considered adequate to build a robust statistical shape model (SSM) covering population variance [5].

**Figure 1:**
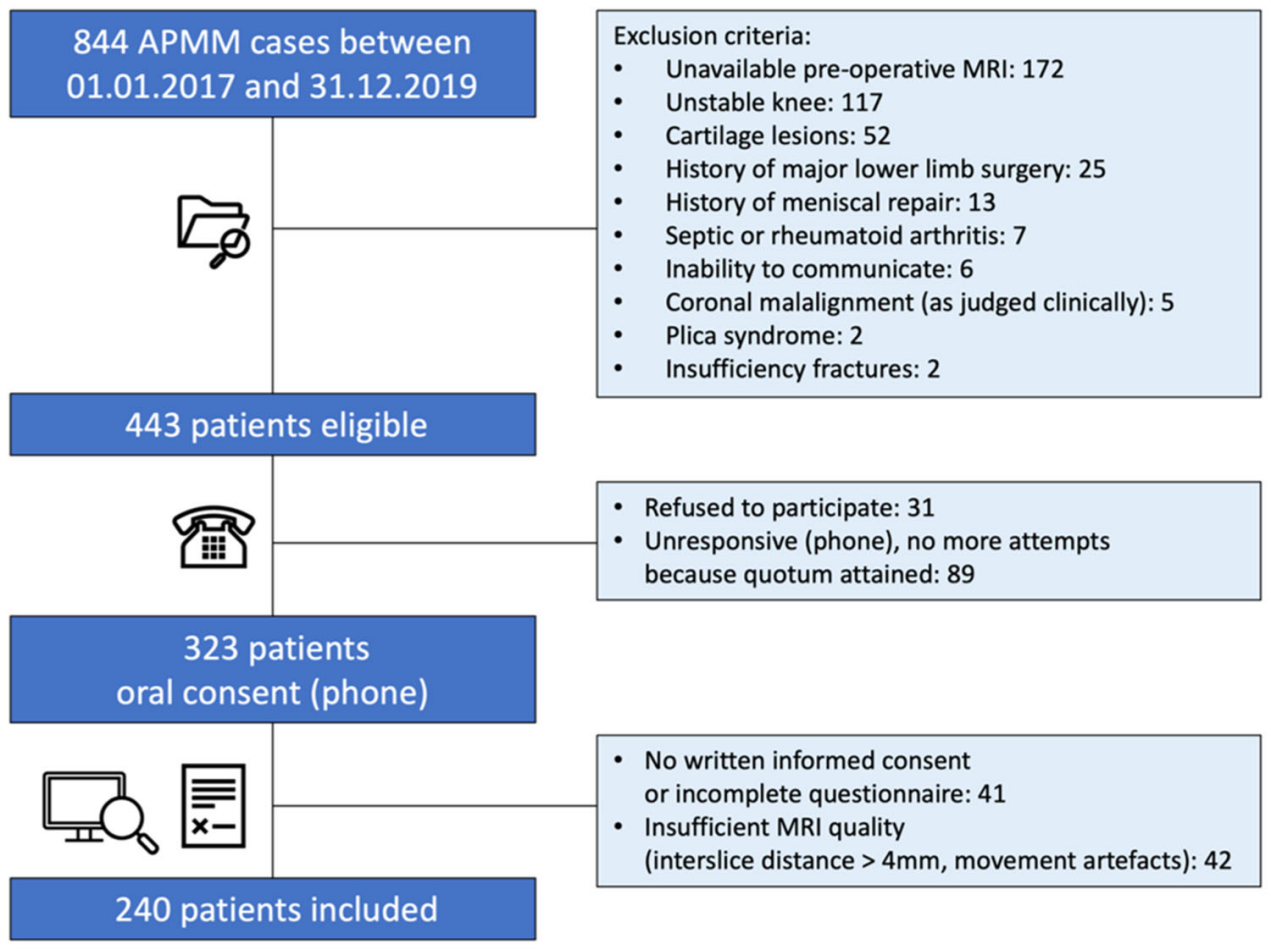
Consolidated Standards of Reporting Trials flow diagram for subject enrolment procedure. All subjects underwent an arthroscopic partial medial meniscectomy (APMM). MRI, magnetic resonance imaging.

The patient selection procedure is summarised in the CONSORT diagram in Figure 1. All patients were first approached by phone. Documents (informed consent and KOOS questionnaire) were sent following oral consent to participate into the study. Upon attaining the predefined sample size in one group (*n* = 120), no additional patients were included in that group, and attempts to contact patients with incomplete KOOS questionnaires or informed consent were stopped when both groups were complete (*n* = 240).

### Data collection

Imaging, demographic and clinical data were collected. The imaging data consisted of the preoperative MRI scans used for diagnosis of the meniscal lesion and was extracted from the hospital picture archiving and communication system (PACS). MRI scans were evaluated by one experienced researcher for motion artefacts and slice thickness < 4 mm. A typical preoperative MRI (1.5 or 3 T) protocol included the following sequences: coronal T1‐weighted (3.5 mm slice thickness, 0.7 × 0.7 mm in‐plane resolution), coronal proton density (PD)‐weighted (3.5 mm slice thickness, 0.5 × 0.5 mm in‐plane resolution), sagittal PD (3.5 mm slice thickness, 0.5 × 0.5 mm in‐plane resolution) and axial T2‐ or intermediate‐weighted (3 mm slice thickness, 0.5 × 0.5 mm in‐plane resolution) images. Collected demographic and clinical data included patient sex, age, weight and height. BMI was calculated from patient weight and height as follows: patient weight (kg) divided by the square patient height (m). Cartilage status (modified Outerbridge classification [3]) was extracted from the surgery reports and verified on the preoperative MRI scans. The KOOS questionnaire [41] at 2 years of follow‐up served as a patient‐reported outcome measure to evaluate response to treatment (RTT).

### Generation of 3D bone models

Preoperative MRI scans were extracted from the PACS system in DICOM format and loaded into Mimics 23.0 (Materialise NV). Following the standard knee scan protocols, sequences in the three perpendicular anatomical planes were available. Using at least two MRI sequences with a perpendicular acquisition plane, distal femur and proximal tibia were segmented into two separate 3D models, each of them consisting of bone and cartilage united. The projected contours of the resulting 3D models were doublechecked on all available sequences and fine‐tuned manually using the ‘Contour edit’ tool of the software package. 3D models of the distal femur and proximal tibia were then saved as triangular meshes and further used to perform the morphological analysis.

### SSM: Data‐driven morphology description

In total, three SSMs were built from the 3D bone models: one separate SSM for the isolated distal femur, one SSM for the isolated proximal tibia [12] and a combined SSM of the tibiofemoral joint in full extension following the methodology described by Audenaert et al. [4].

**Figure 2:**
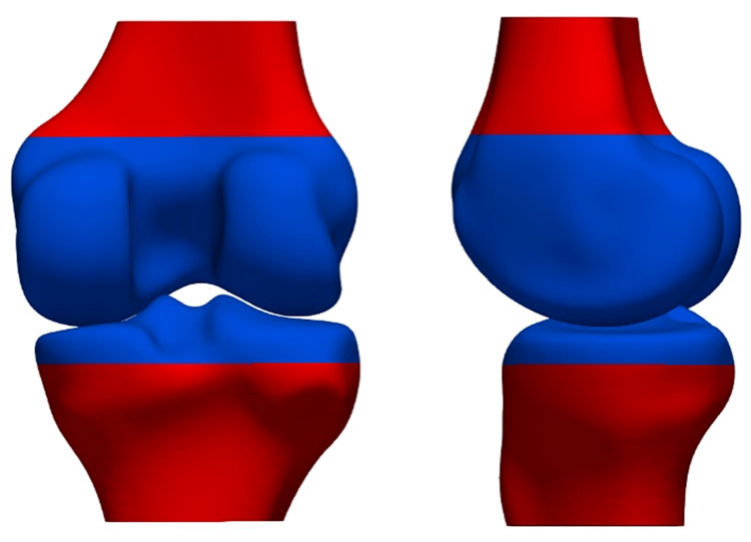
Definition of the distal femur and proximal tibia to be included in the shape analysis (blue region). The red surface region was neglected in the shape analysis. Left: posterior view. Right: medial view.

The first step in building an SSM is the establishment of anatomical correspondences between the patient‐specific bone shapes. This involves the automatic identification and matching of the same anatomical landmarks and regions across the different bone shapes in the data set. Practically, this was achieved through an iterative process of rigid and elastic deformations as described and validated by Danckaers et al. [12]. Briefly, a template triangular bone mesh of the distal femur or proximal tibia was gradually deformed to the bone shape of the patients, resulting in a mesh with a dense set of pseudo‐landmarks that share a consistent anatomical meaning across all patients. Next, all patient bone shapes were rigidly aligned (rotations, translations) to the same position by minimising the sum of distances between all corresponding points. To reduce any variability induced by the MRI scan field of view, only the distal portion of the femur and proximal portion of the tibia was selected for further analysis (blue region in Figure 2).

The previously established anatomical correspondences ensure that the same anatomical region is selected for all patients.

**Figure 3:**
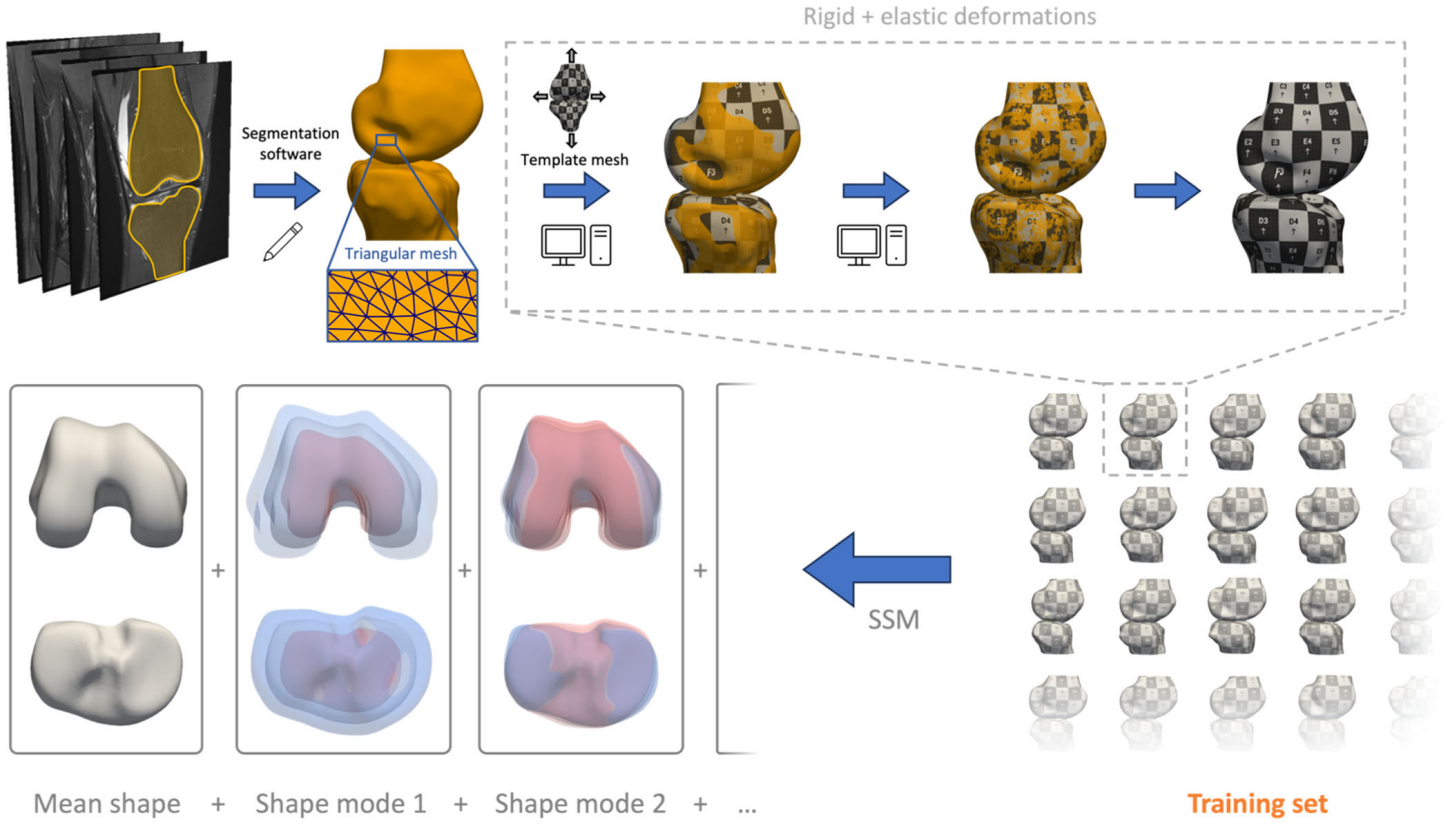
Starting from the medical images, three‐dimensional models were manually created in the segmentation software. Next, anatomical correspondences are computed by rigidly and elastically deforming a template mesh (visualised with checker pattern). These deformed template meshes (=training set) are then further used to construct the statistical shape model (SSM).

A custom Python script was written to construct the SSM. The mean bone shape was calculated, and principal component analysis (PCA) resulted in the main modes of shape variation [11]. PCA is a commonly used mathematical method for dimensionality reduction, which transforms the original variables (numerous 3D model point coordinates *x*, *y* and *z* for each bone shape) linearly into a set of new variables (confined number of modes of shape variation), ordered by decreasing magnitude (explained shape variance). Together, the mean bone shape and the modes of shape variation define the SSM, and they can describe any shape of the same nature as a weighted sum of those modes. The weight factors for each mode of shape variation are then called principal component (PC) weights. Given the SSM, the PC weights for each mode of shape variation are then used to characterise the patient‐specific bone morphology. The SSM construction is summarised and illustrated in Figure 3.

In the combined SSM, the adopted method of Audenaert et al. [4] realigned the individual bones to an average (neutral) relative position before inclusion in the SSM, thereby removing any potential relative positional information (e.g., induced by patient positioning in the scanner). Without this realignment, any positional variation (e.g., flexion/extension, varus/valgus, internal/external rotation) would also be captured in the main modes of shape variation, thereby resulting in a less compact SSM.

For all three SSMs in this study, the modes of shape variation were defined on the complete data set of 240 knees. Model compactness and generalisation were evaluated as performance metrics for all three SSMs [5].

#### Model compactness

Compact shape models can describe any new shape instance with as little modes of shape variation as possible [16]. SSM compactness is described as the cumulative explained variance in the function of the number of modes. The higher this value, the fewer modes are needed for the SSM to describe shape variation at the population level and increase model performance. A cumulative explained variance ratio of more than 98% was aimed for when choosing the appropriate number of modes.

#### Model generalisability

For matters of future clinical applications, the SSM should generalise to unseen shapes of the same nature [16]. That is, it should be able to describe unseen bone shapes to a certain level of accuracy. Repeated leave‐one‐out cross‐validation experiments were performed in order to assess this capability for an increasing number of modes of shape variation. The generalisation metric was then defined as the average description error (root mean square error [RMSE]) over all experiments.

#### SSM in relation to clinical data and RTT

PC weights for the specific modes of shape variation were calculated for all patients in both R and MPMS groups. The PC weights for the first three modes of shape variation were then compared between the R and MPMS group, and a physical meaning was assigned to those modes (results section Knee morphology comparison between R and MPMS group). In the next step, correlations between demographic, clinical and morphology variables (PC weights) were assessed (section Correlation analysis between demographic, clinical and morphology variables). Finally, a predictive algorithm for RTT based on knee morphology was trained and evaluated (section Prediction of RTT based on knee morphology).

### Statistical analysis

All statistical analyses were performed in RStudio (Version 2022.07.0.1; R Studio, PBC) and R (version 4.2.1; R Foundation). Training and cross‐validation of the predictive algorithm were performed in Python (open‐source library scikit‐learn v1.1.2) [34]. Statistical significance level was defined at *p* < 0.05.

#### Study population

Descriptive statistics for the continuous variables patient age, weight, height and BMI included mean and standard deviation, while the categorical variable patient sex was reported as count and percentage. Differences in distributions between the R and MPMS groups were tested by the two‐sided student *t* test for the continuous variables, Fischer's exact test for the variable sex and a *χ* ^2^ test of independence for cartilage status.

#### Knee morphology comparison between R and MPMS group

PC weights distributions for the first three modes of shape variation were compared between the R and MPMS groups using Welch's unequal variances *t* test.

#### Correlation analysis

Pearson correlation coefficients were calculated between the following variables: patient sex (recoded as 0 for female and 1 for male), patient age, patient length, patient weight, KOOS and the PC weights of the first three modes of shape variation for all three SSM. Statistical significant Pearson correlation coefficients were reported in a correlation matrix.

#### Prediction of RTT

The predictive value of PC weights for RTT (recoded as 1 for the R group and 0 for the MPMS group) was assessed. Prediction of RTT was performed by means of logistic regression on the PC weights in a leave‐one‐out cross‐validation experiment. Sensitivity and specificity were calculated for the detection of both RTT and MPMS [39]. In addition, the area under the curve for the receiver operating characteristic curve [39] (AUC‐ROC) was calculated. A bootstrapping experiment with 1000 iterations was performed to calculate 95% confidence intervals (CIs).

## RESULTS

### Study population

**Figure 4:**
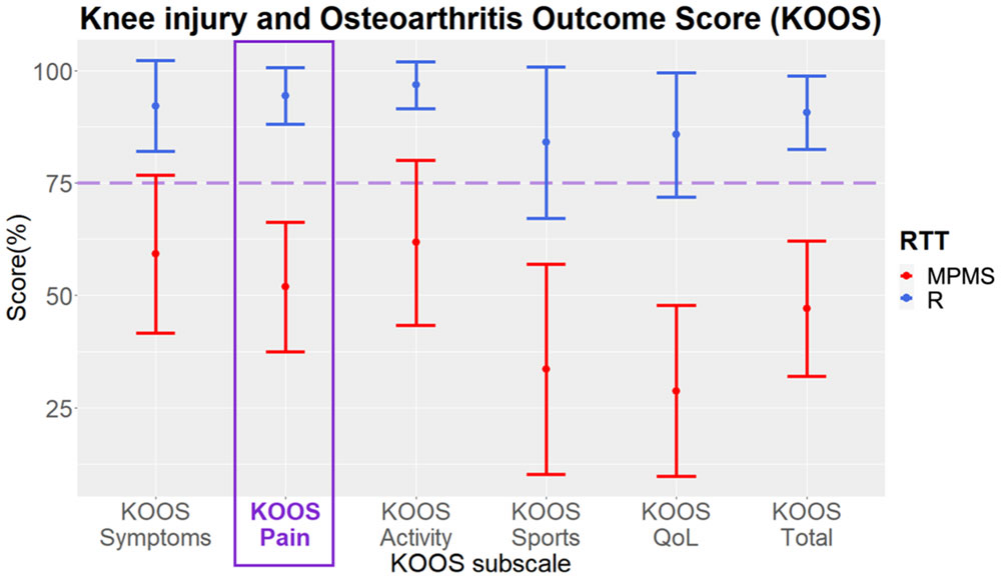
Mean ± standard deviation of all Knee injury and Osteoarthritis Outcome Score (KOOS) subscales for both medial postmeniscectomy syndrome group (MPMS) (red) and response to treatment group (R) (blue) groups. RTT, response to treatment.

There were no significant differences between the R and MPMS groups in patient sex, patient age, patient weight, patient height and BMI distributions (Table 1). Cartilage status according to the modified Outerbridge scale (intraoperative assessment) was not significantly different between the two groups for all regions. The KOOS and all of its subscales were significantly different (*p* < 0.001 for all KOOS subscales, Figure 4).

**Table 1:** Count and percentage for patient sex and cartilage lesion classification (modified Outerbridge), mean ± SD for variables patient age, patient weight, patient height and BMI in both R and MPMS groups.

	R group	MPMS group	*p* Value
Patient sex [number (%)]^a^	29 (24.2) females, 91 (75.8) males	36 (30.0) females, 84 (70.0) males	n.s.
Patient age (years)^b^	50.7 ± 12.1	52.7 ± 10.5	n.s.
Patient weight (kg)^b^	82.0 ± 15.1	82.2 ± 14.4	n.s.
Patient height (cm)^b^	177.2 ± 8.6	175.2 ± 9.6	n.s.
BMI (kg/m^2^)^b^	26.0 ± 3.8	26.7 ± 3.4	n.s.
Cartilage lesion classification [number (%)]^c^			
Medial femoral condyle			
Grade 0–I	47 (39.2)	37 (31.4)	n.s.
Grade II	50 (41.7)	43 (36.4)	
Grade III	17 (14.1)	32 (27.1)	
Grade IV focal	6 (5.0)	6 (5.1)	
Medial tibial plateau			
Grade 0–I	56 (46.7)	57 (48.3)	n.s.
Grade II	50 (41.6)	46 (39.0)	
Grade III	11 (9.2)	13 (11.0)	
Grade IV focal	3 (2.5)	2 (1.7)	
Lateral femoral condyle			
Grade 0–I	71 (59.2)	85 (72.0)	n.s.
Grade II	47 (39.2)	29 (24.6)	
Grade III	1 (0.8)	1 (0.9)	
Grade IV focal	1 (0.8)	3 (2.5)	
Lateral tibial plateau			
Grade 0–I	70 (58.3)	85 (72.0)	n.s.
Grade II	47 (39.2)	29 (24.6)	
Grade III	1 (0.8)	2 (1.7)	
Grade IV focal	2 (1.7)	2 (1.7)	

Abbreviations: BMI, body mass index; MPMS, medial postmeniscectomy syndrome; n.s., nonsignificant.

^a^ Fischer exact test.

^b^ Two‐sided student *t* test.

^c^ *χ* ^2^ test of independence.

### Knee morphology comparison between R and MPMS groups

#### Distal femur

Regarding compactness, the SSM of the distal femur captured 98.0% of the shape variance in the first 36 modes of shape variation (model compactness). Generalisation error to unseen distal femur shapes in leave‐one‐out experiments was on average 0.30 mm (RMSE). More detailed performance metrics of the SSMs can be found in Supporting Information.

**Figure 5:**
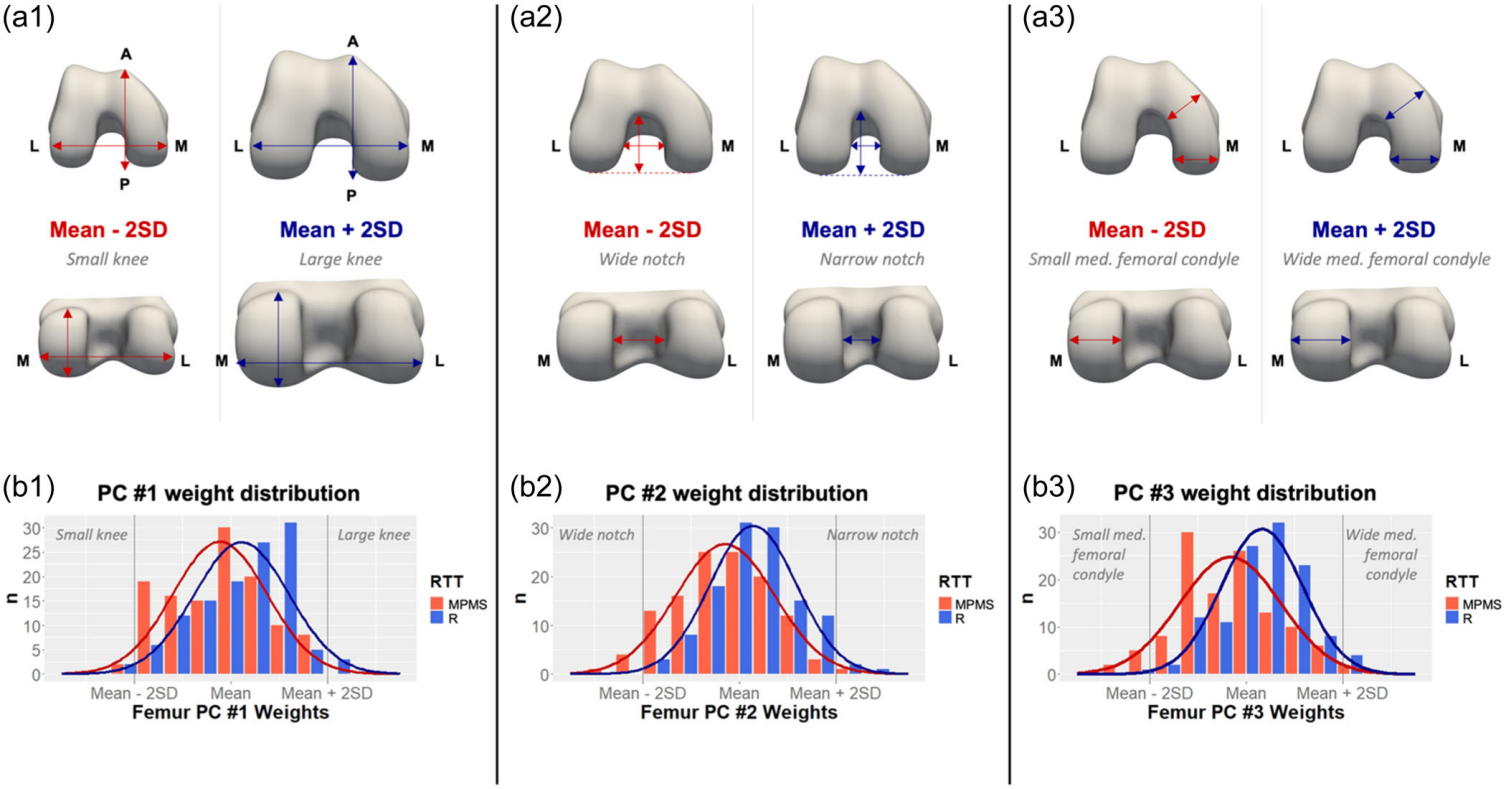
(a1–a3) First three modes of shape variation in distal femur: (top) inferior view, (bottom) posterior view. (b1–b3) Histogram of principal component (PC) weights distribution for the first three modes of shape variation in medial postmeniscectomy syndrome group (red) and response to treatment group (R) (blue) group. A, anterior; P, posterior; L, lateral; M, medial; MPMS, medial postmeniscectomy syndrome group; *n*, number of cases; RTT, response to treatment; SD, standard deviations.

The first mode of shape variation is the 3D size of the distal femur (Figure 5, left column). Distal femora of the MPMS group were significantly smaller than those in the R group (*p* < 0.001). The second mode of shape variation (Figure 5, middle column) captured the mediolateral (ML) intercondylar notch width. The MPMS group had a significantly wider intercondylar notch (*p* < 0.001). The third mode of shape (Figure 5, right column) variation encompassed the ML width of the medial femoral condyle and anteroposterior (AP) length of both femoral condyles. MPMS knees had a significantly smaller ML medial femoral condyle and larger AP femoral condyles (*p* < 0.001). Together, these three modes of shape variation accounted for 86.7% of the total femoral shape variance.

#### Proximal tibia

The SSM of the proximal tibia described 98.1% of the shape variance in the first 37 modes of shape variation that. Leave‐one‐out experiments resulted in an average generalisability error of 0.25 mm RMSE.

**Figure 6:**
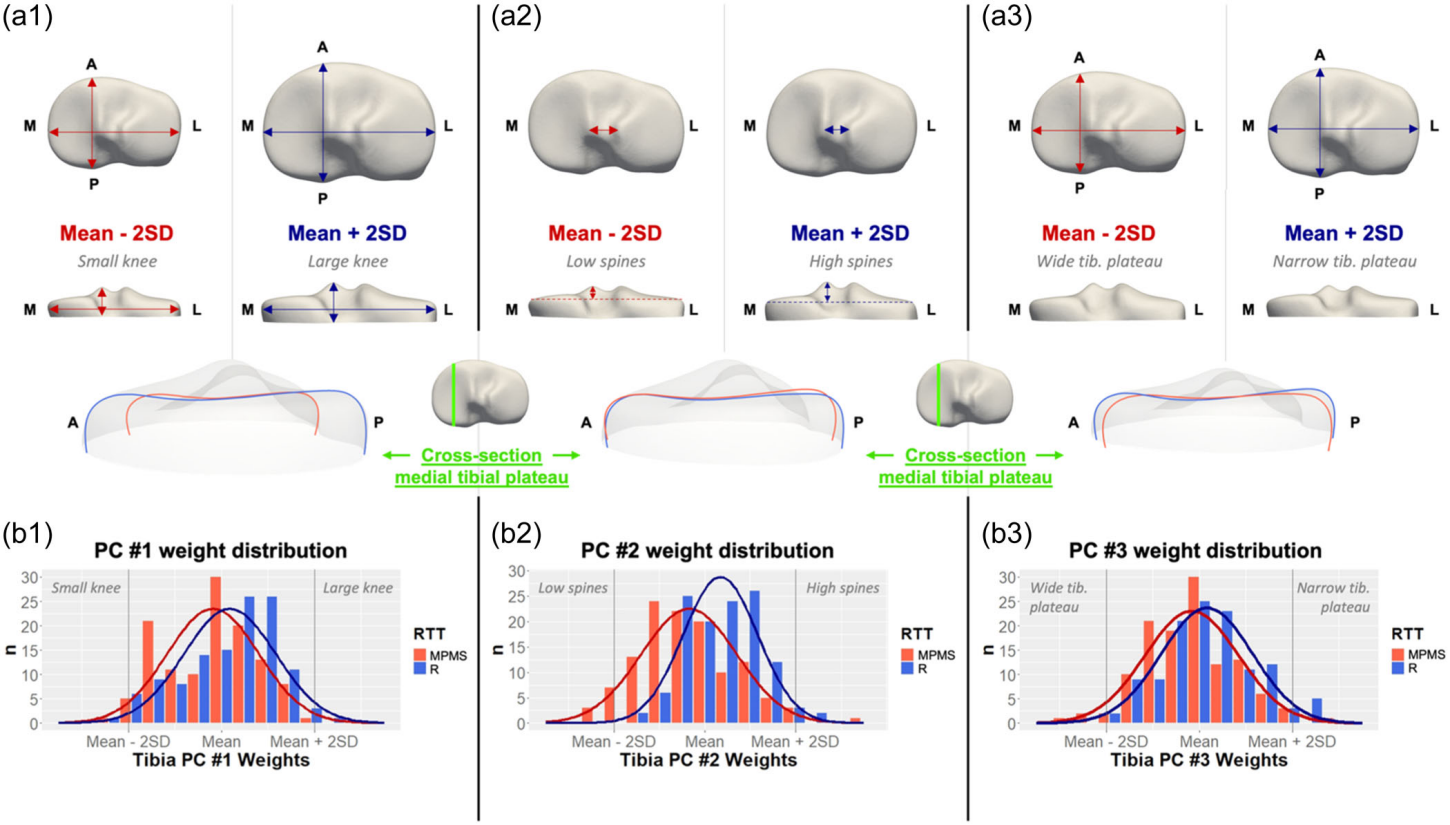
(a1–a3) First three modes of shape variation in proximal tibia: (top) superior view, (middle) posterior view, (bottom) cross‐sectional medial view of the medial tibial plateau. (b1–b3) Histogram of principal component (PC) weights distribution for the first three modes of shape variation in medial postmeniscectomy syndrome group (MPMS) (red) and response to treatment group (R) (blue) group. A, anterior; L, lateral, M, medial; *n*, number of cases, P, posterior; RTT, response to treatment; SD, standard deviations.

The first mode of shape variation (Figure 6, left column) in the proximal tibia was 3D size. The MPMS group consisted of significantly smaller tibial plateaus (*p* = 0.005). The second mode of shape variation (Figure 6, middle column) included ML width of the tibial plateau and interspine distance, as well as tibial spine height variation. The MPMS group had a significantly wider interspine distance and lower tibial spines (*p* < 0.001). The third mode of shape variation (Figure 6, right column) included the AP length of the tibial plateau and the sagittal concavity of the medial tibial plateau. A small difference was observed between the R and MPMS groups for this mode of shape variation (*p* = 0.01), where tibial plateaus from the MPMS group had a more pronounced ML width relative to their AP depth and a less concave (sagittal plane) medial tibial plateau. Together, these three modes of shape variation explained 83.7% of the total tibial shape variance.

#### Tibiofemoral joint (combined shape model in neutral position)

The combined SSM of femur and tibia together captured 98.0% of the variance in 46 modes of shape variation as a measure of the model compactness. This corresponded to a generalisation error of 0.34 mm in the leave‐one‐out experiments.

**Figure 7:**
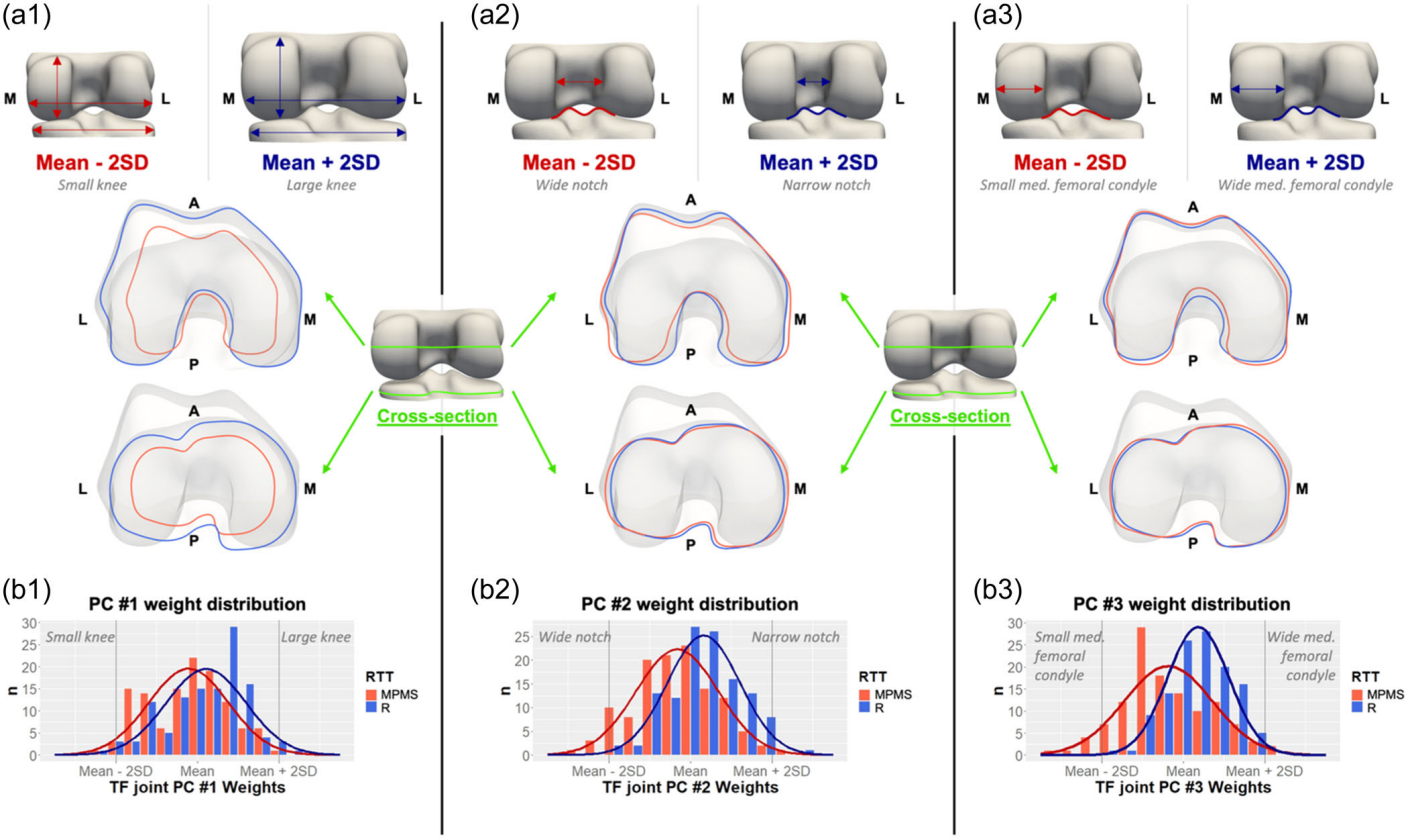
(a1–aA3) First three modes of shape variation in tibiofemoral joint: (top) posterior view, (middle) inferior view cross‐sections of distal femur, (bottom) inferior view cross‐sections of proximal tibia. (b1–b3) Histogram of principal component (PC) weights distribution for the first three modes of shape variation in medial postmeniscectomy syndrome group (MPMS) (red) and response to treatment group (R) (blue) group. A, anterior; L, lateral, M, medial; *n*, number of cases; P, posterior; RTT, response to treatment; SD, standard deviations.

The main mode of shape variation (Figure 7, left column) in the tibiofemoral joint was size and accounted for 83.4% of the total shape variance. The distribution of the PC weights was significantly shifted towards smaller knees in the MPMS group (*p* < 0.001). The second mode of shape variation (Figure 7, middle column) described the intercondylar ML notch width and tibial interspine distance. A wider femoral intercondylar notch and larger tibial interspine distance were observed in the MPMS group (*p* < 0.001). The third mode of shape variation (Figure 7, right column) included the ML width of the medial femoral condyle and the height of the tibial spines. A significantly smaller medial femoral condyle and less pronounced tibial spines were observed in the MPMS knees (*p* < 0.001). Together, these three modes of shape variation described 88.2% of the total tibiofemoral shape variance.

### Correlation analysis between demographic, clinical and knee morphology variables

Statistical significant Pearson correlation coefficients (*R*²) were summarised in a correlation matrix (Table 2) for the demographic, clinical and morphological variables of the distal femur, proximal tibia and tibiofemoral joint. RTT was encoded as 0 for the MPMS group and 1 for the R group. RTT was strongly positively correlated with all KOOS subscores (*p* < 0.001). For all three SSMs (femur, tibia and tibiofemoral joint), the PC weights of the first three modes of shape variation were all very weakly to weakly (*R*² between 0.16 and 0.37) correlated with RTT (*p* ≤ 0.01). None of the demographic or clinical variables were correlated with RTT.

**Table 2:** Correlation matrix of demographic, clinical, PROM‐ and SSM‐derived variables.

	RTT	Sex	Age	Length	Weight	BMI	KOOS symptoms	KOOS pain	KOOS activity	KOOS sports	KOOS QoL	KOOS total	Femur PC #1 weights	Femur PC #2 weights	Femur PC #3 weights	Tibia PC #1 weights	Tibia PC #2 weights	Tibia PC #3 weights	Tibiofemoral joint PC #1 weights	Tibiofemoral joint PC #2 weights	Tibiofemoral joint PC #3 weights
RTT	1.00						0.74	0.87	0.78	0.77	0.85	0.86	0.23	0.29	0.33	0.18	0.35	0.16	0.22	0.32	0.37
Sex		1.00	−0.19	0.70	0.55	0.21				0.20		0.14	0.77	−0.21		0.79	−0.13		0.78	−0.20	
Age			1.00	−0.32						−0.15		−0.13	−0.21	−0.23		−0.19			−0.20	−0.22	
Length				1.00	0.67	0.14	0.17	0.14		0.25		0.17	0.80			0.79			0.80		
Weight					1.00	0.82							0.63		−0.14	0.62			0.63		−0.17
BMI						1.00			−0.13				0.24		−0.14	0.24			0.24		−0.15
KOOS symptoms							1.00	0.84	0.82	0.76	0.81	0.90	0.26	0.21	0.31	0.23	0.23		0.25	0.24	0.32
KOOS pain								1.00	0.93	0.82	0.90	0.96	0.26	0.28	0.35	0.21	0.31	0.15	0.25	0.31	0.38
KOOS activity									1.00	0.82	0.86	0.94	0.25	0.26	0.32	0.22	0.31	0.14	0.24	0.29	0.35
KOOS sports										1.00	0.83	0.92	0.33	0.19	0.27	0.31	0.25		0.33	0.22	0.29
KOOS QoL											1.00	0.95	0.24	0.27	0.35	0.19	0.36	0.17	0.23	0.31	0.40
KOOS total												1.00	0.29	0.26	0.34	0.25	0.32	0.15	0.28	0.29	0.37
Femur PC #1 weights													1.00			0.98			1.00		
Femur PC #2 weights														1.00			0.32	0.46		0.99	
Femur PC #3 weights															1.00		0.16				0.93
Tibia PC #1 weights																1.00			0.99		
Tibia PC #2 weights																	1.00			0.40	0.40
Tibia PC #3 weights																		1.00		0.49	−0.18
Tibiofemoral joint PC #1 weights																			1.00		
Tibiofemoral joint PC #2 weights																				1.00	
Tibiofemoral joint PC #3 weights																					1.00

*Note*: Only significant (*p* < 0.05) Pearson *R*² correlation coefficients were plotted.

Abbreviations: BMI, body mass index; KOOS, Knee injury and Osteoarthritis Outcome Score; PC, principal component; PROM, patient‐reported outcome measure; RTT, response to treatment; SSM, statistical shape model.

Patient sex, length and weight were strongly positively correlated (*R*² between 0.62 and 0.80) with the first mode of shape variation (3D size) for all three SSMs (*p* < *0.001*). Patient sex showed a very weak and weak negative correlation with the second mode of shape variation (femoral notch width and tibial interspine distance) from, respectively, the SSM of the proximal tibia (*R* ^2^ = −0.13, *p* = 0.047) and the SSM of distal femur and tibiofemoral joint (*R* ^2^ between −0.21 and 0.20, *p* < 0.002).

### Prediction of RTT based on knee morphology

**Figure 8:**
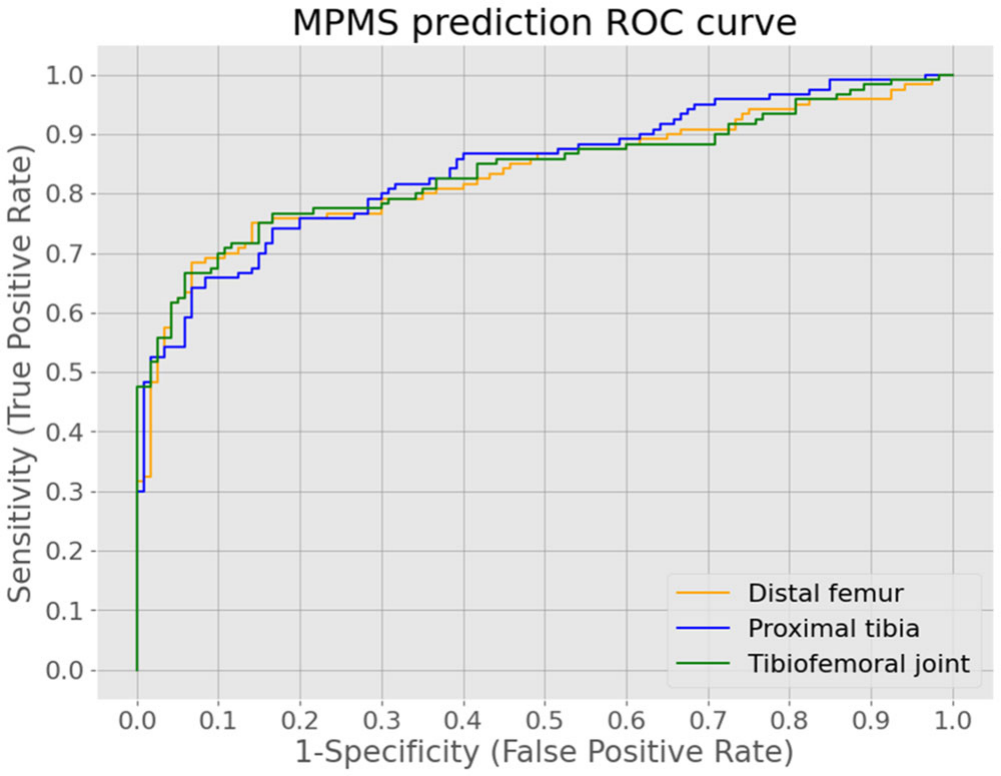
Receiver operating characteristic curves for three distinct classifiers, using a set of distal femur, proximal tibia or tibiofemoral joint shape features as predictor for medial postmeniscectomy syndrome (MPMS). ROC, receiver operating characteristic.

Distal femur morphology is an independent predictor for RTT. Solely based on the distal femur PC weights, MPMS was predicted with a sensitivity of 74.8% (95% CI: 74.3%–75.3%) and a specificity of 80.3% (95% CI: 79.8%–80.8%). AUC‐ROC (Figure 8, yellow curve) for this classifier was 0.827 (95% CI: 0.824–0.830).

The predictive logistic regression algorithm with proximal tibia PC weights as input, identified MPMS in a leave‐one‐out cross‐validation experiment with a sensitivity of 74.7% (95% CI: 74.2%–75.2%) and a specificity of 78.5% (95% CI: 78.0%– 79.0%). AUC for the ROC curve (Figure 8, blue curve) was 0.836 (95% CI: 0.833–0.839) for this classifier based on proximal tibia morphology.

Tibiofemoral joint morphology predicted MPMS with a sensitivity of 74.9% (95% CI: 74.4%–75.4%) and a specificity of 81.0% (95% CI: 80.6%–81.5%) in a leave‐one‐out cross‐validation experiment. AUC‐ROC was 0.840 (95% CI: 0.837–0.843) for this tibiofemoral joint morphology‐based classifier. The ROC curve for the classifier using a set of tibiofemoral joint shape features is shown in Figure 8 (green curve).

## DISCUSSION

This study unveils for the first time bony knee morphological differences between responders to APMM and MPMS patients: a smaller overall size of the knee, a wider intercondylar notch and a smaller medial femoral condyle were the main morphological variations identified in MPMS knees. Moreover, as a second key finding in this study, morphology‐based predictive models demonstrated a sensitivity of more than 75% and a specificity surpassing 80% in anticipating the outcome of APMM.

Based on these findings, a biomechanical mechanism is hypothesised to explain the main differentiating mode of shape variation between responders to APMM and MPMS. While often excluded or filtered out in statistical shape analysis, a significant difference in knee size was observed between the R and MPMS group knees for the three SSMs: isolated femur, isolated tibia and combined in the tibiofemoral joint. The knees from the MPMS group patients were significantly smaller than their counterparts in the R group, in contrast with no differences in patient length, weight and BMI between both groups. A smaller knee implicates a smaller articular contact surface area. In biomechanics, pressure is defined as force per unit area of surface. Therefore, a smaller knee loaded with a similar body weight is subject to higher pressure. Different studies already established increased contact pressure as a central driver in progressive knee degeneration [30, 33].

To the authors' knowledge, this is the largest 3D knee morphology database of post‐meniscectomized patients, consisting of imaging, demographic and clinical (incl. patient‐reported outcome measure) data at 2 years postoperatively. Similar techniques based on statistical shape modelling have already been applied on other pathologies, for example, for automatic staging of trochlear dysplasia [10] or identifying morphological bone variations linked to scaphoid fractures [8].

Previously, our pilot study on medial compartment degeneration already identified the small medial femoral condyle morphotype [17] and its potential association with early medial compartment degeneration. Several in silico and in vitro studies have confirmed the load‐distributing function of the meniscus, as well as the increase in cartilage peak stress after partial meniscectomy [43]. The smaller contact surface area in the small medial femoral condyle morphotype could already imply a higher peak stress compared to a wide medial femoral condyle knee. By further reducing the contact surface area, meniscus functional loss by degeneration or APMM might additionally increase the peak stress, leading to insufficiency of the cartilage and subchondral bone to bear the load [27]. This is clinically reflected in the postmeniscectomy syndrome.

Several studies established the association between coronal alignment of the knee and pathology [22, 31]. In our study, the influence of alignment as a latent prognostic factor was minimised since all knees had a coronal alignment within the physiological range per clinical judgement. The combination of a full lower limb phenotype analysis with this confined knee morphology analysis might even result in more robust algorithms to predict APMM outcomes, irrespective of knee malalignment.

A clustering analysis by Hohlmann et al. [23] revealed no distinct morphotypes in end‐stage knee osteoarthritis (OA) knees. In contrast to their conclusion, our current study found that knee shape (including size and aspect ratio) is highly correlated with patient demographics (sex, length, weight) and even APMM outcome. These contradictory findings can be explained by the fact that unsupervised clustering algorithms are typically less powerful for classification purposes. Importantly, the previous authors corrected for both size and aspect ratio, while these parameters were found to be significantly correlated to several clinical variables in the current study.

Based on SSMs, Bowes et al. created a score to quantify radiological disease progression in cases from the Osteoarthritis Initiative database [9, 28]. Tack et al. [45] extended this concept further to a set of OA biomarkers by adding traditional measurements (e.g., volume and surface area) and SSM‐derived features for femoral bone, tibial bone and medial and lateral meniscus. This set of biomarkers proved superior performance for the prediction of total knee replacement within 1 year in comparison with the bony SSM‐derived features alone. The aforementioned studies support the hypothesis of bone shape as a biomarker and predictor for wear‐related pathology. Indeed, the postmeniscectomy syndrome also lies in the spectrum of degenerative knee pathologies, and this current study was able to predict its onset solely based on the knee morphology.

The relevance of this work lies potentially in the health‐economical aspect of medial meniscus lesion treatment [21, 49]. This is a promising first step towards a better identification of candidate patients for APMM [35]. Smarter risk definitions and patient selection in the future will result in more personalised health care [47]. Eventually, forthcoming clinical decision support systems might serve as a tool for clinicians to help achieving this aim.

The strengths of this study include the multicentre study design, the high volume of manually segmented MRI scans, as well as the unbiased methodology to describe knee morphology quantitatively. Instead of using predefined measurements, patterns of morphological variation were extracted by a robust mathematical algorithm. Furthermore, this high level of automated analysis starting from the 3D models of femur and tibia allows the analysis of even larger databases at a low marginal time cost (even zero human marginal time cost). Another strength of this unique data set is the highly homogenous group of patients and the equal distribution of collected potential latent demographic variables (patient sex, height and weight) influencing knee morphology across both R and MPMS groups. Finally, the preoperative imaging used to create the 3D models is already available for this pathology, even in a standard clinical setting.

This study shows promising results, despite some limitations. First, because of the multicentre retrospective study design, no precise data were available on possibly confounding variables such as personalised medication and rehabilitation protocol. However, the clinical centres (in Antwerp, Milan and Regensburg) have closely matched patient demographics and healthcare systems. All patients received nearly identical postoperative care, adhering to the latest clinical guidelines.

Second, during the follow‐up period after APMM, no new MRI scans were performed unless clinically necessary. As a result, quantitative standardised measurements of the resected meniscal tissue could not be calculated, nor retrieved from the surgery report, and no subgroup analysis based on the presence of radiologic signs such as bone marrow oedema, osteonecrosis or meniscal retear could be performed.

Finally, it should be noted that only patients with a clinically normal coronal malalignment were included in this study, per surgeon clinical assessment. In a real‐world clinical protocol for meniscus lesion management, no full‐leg standing radiographs, nor full‐leg computerised tomography scans, are indicated for these patients. Given the retrospective nature of this study, exact coronal alignment measurements were, therefore, unavailable.

Future research includes large prospective validation studies, with additional follow‐up imaging, to warrant the generalisability of the findings in this study.

In conclusion, morphological variations determine the clinical outcome of APMM in a patient population with comparable weight, height, alignment and cartilage status. More specifically, a smaller total knee size, a wider intercondylar notch and a smaller medial femoral condyle are strongly linked with postmeniscectomy syndrome. In addition, a predictive model was able to anticipate the clinical outcome of APMM at 2 years of follow‐up with a sensitivity above 75% and a specificity exceeding 80%.

## AUTHOR CONTRIBUTIONS

Peter Verdonk conceived of the presented idea. The concept was further thought through in cooperation with Annemieke Van Haver, Femke Danckaers, Jan Sijbers and Jonas Grammens. Kristien Vuylsteke, Jonas Grammens, Lotem Mahluf, Peter Angele, Elizaveta Kon and Peter Verdonk designed the data collection process. The computational framework was developed by Femke Danckaers and Jonas Grammens. Jonas Grammens and all members of the MEFISTO WP1 group contributed to the data collection and preprocessing of raw imaging data into accurate 3D models. Femke Danckaers and Jan Sijbers verified the analytical methods. Peter Verdonk, Peter Angele and Elizaveta Kon supervised the clinical findings of this work. All authors discussed the results and contributed to the final manuscript.

## MEFISTO WP1 GROUP

Maoz Shemesh and Eran Linder‐Ganz (Active Implants BV, Haarlem, Netherlands); Alexander Stockmann and Girish Pattappa (Laboratory for Experimental Trauma Surgery, Department of Trauma Surgery, University Medical Center Regensburg, Regensburg, Germany); Denitsa Docheva (Department of Musculoskeletal Tissue Regeneration, Orthopaedic Clinic König‐Ludwig‐Haus, University of Wurzburg, Germany); Gino Mestach (Antwerp Surgical Training, Anatomy and Research Centre, University of Antwerp, Antwerp, Belgium); Daniele Altomare (IRCCS Humanitas Research Hospital, Rozzano, Milan, Italy; Department of Biomedical Sciences, Humanitas University, Pieve Emanuele, Milan, Italy).

## CONFLICT OF INTEREST STATEMENT

The authors declare no conflict of interest.

## ETHICS STATEMENT

This study was performed in line with the principles of the Declaration of Helsinki. Approval was granted by the Ethics Committee of University Hospital Antwerp (21 October 2019/No. B300201941743), University of Regensburg (11 December 2019/No. 19‐1621‐101) and IRCCS Instituto Clinico Humanitas (29 April 2020/No. 2515). Informed consent was obtained from all patients prior to their inclusion.

## Supporting information

Supporting information.

## Data Availability

The data sets used and/or analysed during the current study are available from the corresponding author on reasonable request.
