# Supplementary material for: Three‐dimensional bone morphology is a risk factor for medial postmeniscectomy syndrome: A retrospective cohort study
Source: J Exp Orthop. 2024 Jul 21;11(3):e12090. doi: 10.1002/jeo2.12090 (PMC11260280; doi:10.1002/jeo2.12090)
Supplement: Supplementary file 1 — Supporting information. [file JEO2-11-e12090-s001.docx]

## Appendix

## Quality metrics SSM distal femur


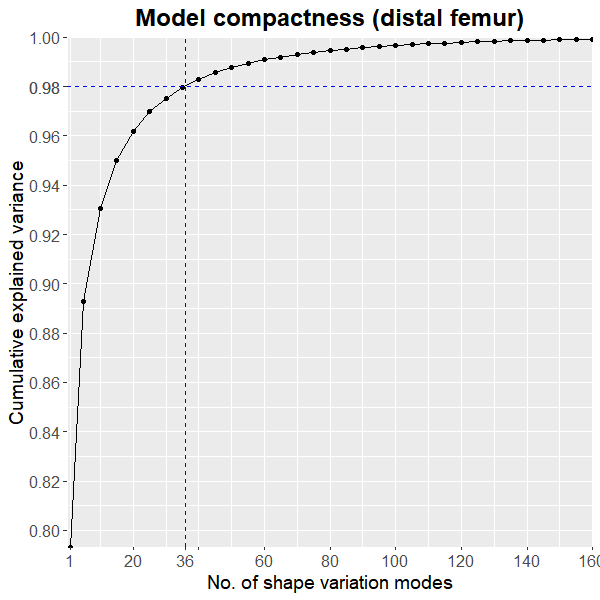


Fig. A.1: Model compactness (% cumulative explained variance) in function of number of shape variance modes for the distal femur SSM.


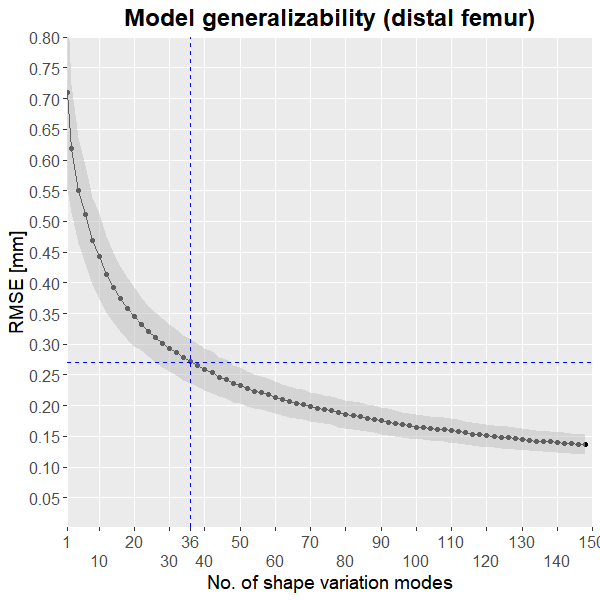


Fig. A.2: Model generalizability (RMSE, mean +/- SD, mm) in function of number of modes of shape variation for the distal femur SSM. Generalization error mean and SD were calculated over all leave-one-out experiments per number of modes that were used to construct the SSM.

## Quality metrics SSM proximal tibia


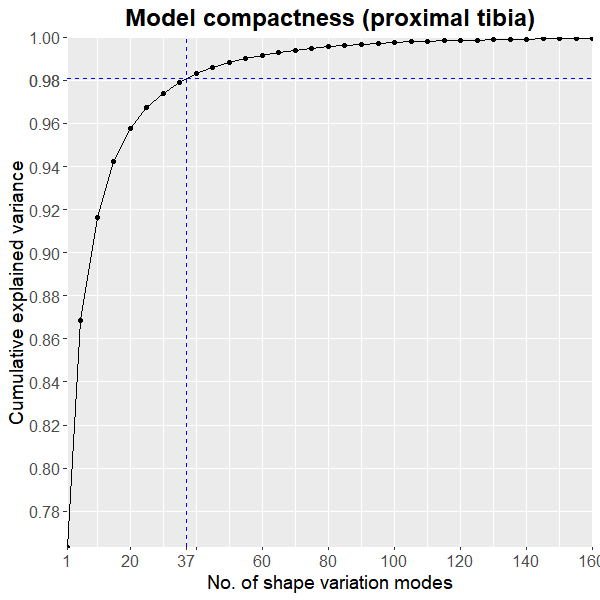


Fig. B.1: Model compactness (% cumulative explained variance) in function of number of shape variance modes for the proximal tibia SSM.


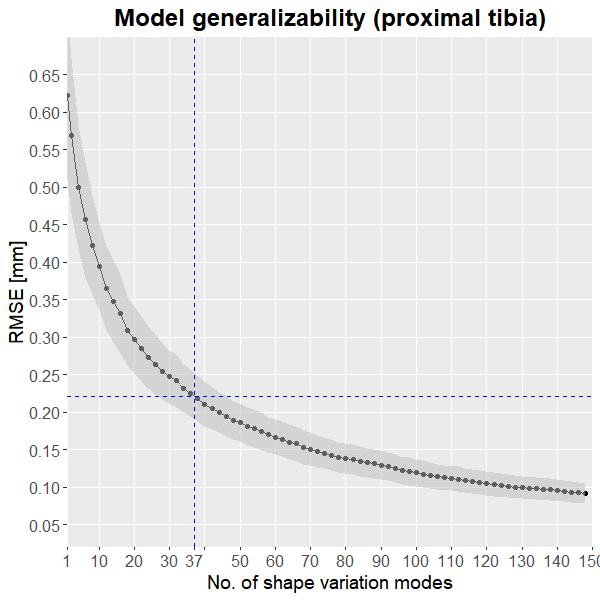


Fig. B.2: Model generalizability (RMSE, mean +/- SD, mm) in function of number of modes of shape variation for the proximal tibia SSM. Generalization error mean and SD were calculated over all leave-one-out experiments per number of modes that were used to construct the SSM.

## Quality metrics SSM tibiofemoral joint


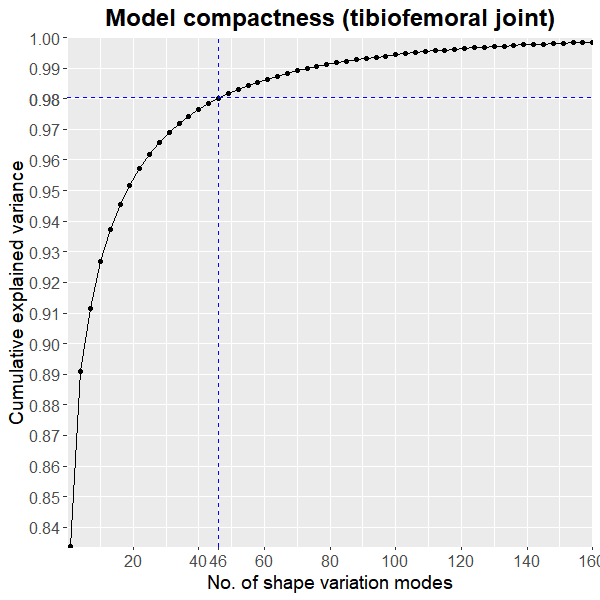


Fig. C.1: Model compactness (% cumulative explained variance) in function of number of shape variance modes for the tibiofemoral joint SSM.


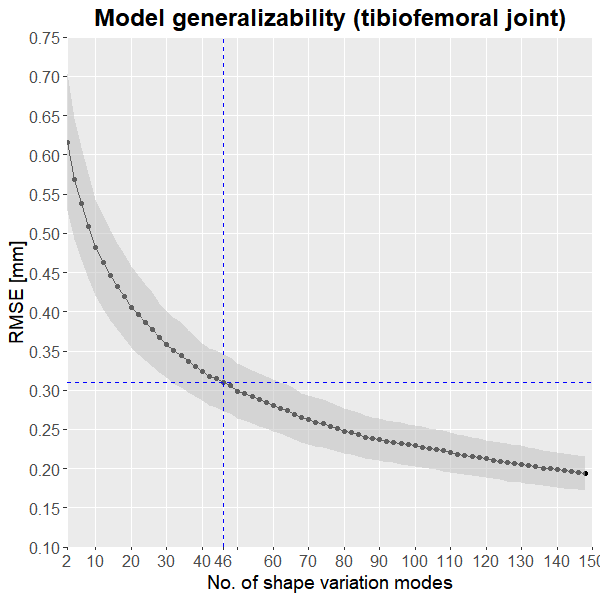


Fig. C.2: Model generalizability (RMSE, mean +/- SD, mm) in function of number of modes of shape variation for the tibiofemoral joint SSM. Generalization error mean and SD were calculated over all leave-one-out experiments per number of modes that were used to construct the SSM.
